# Improving Mass-Rearing Techniques for Releases of *Floracarus perrepae*, a Biological Control Agent for Old World Climbing Fern (*Lygodium microphyllum*)

**DOI:** 10.3390/insects16020135

**Published:** 2025-01-31

**Authors:** Jessene Aquino-Thomas, Logan Crees, Michelle Miles, Melissa C. Smith, Ellen C. Lake, F. Allen Dray Jr.

**Affiliations:** 1 USDA-ARS Invasive Plant Research Laboratory, 3225 College Avenue, Fort Lauderdale, FL 33314, USA; logan.crees@usda.gov (L.C.); michelle.miles@usda.gov (M.M.); melissa.smith@usda.gov (M.C.S.); allen.dray@usda.gov (F.A.D.J.); 2 Mt. Cuba Center, 3120 Barley Mill Road, Hockessin, DE 19707, USA; elake@mtcubacenter.org

**Keywords:** biological control, eriophyid mite, *Floracarus perrepae*, invasive species, *Lygodium microphyllum*, mass-rearing

## Abstract

The United States Department of Agriculture—Invasive Plant Research Laboratory started limited production of a biological control mite, *Floracarus perrepae*, in 2008 for release against the invasive fern *Lygodium microphyllum*. Mass-rearing and release of the biological control agent was initiated in 2014 as part of the Comprehensive Everglades Restoration Plan to address the challenge of low establishment rates observed from 2008 to 2010. In late 2021, we critically analyzed our rearing protocols, focusing on aging galls and increasing plant vigor. These adjustments resulted in an exponential increase in colony productivity. We implemented bi-weekly monitoring of mite numbers within galls and identified the gall age class with the highest mite density. Based on this information, we developed a systematic method involving weekly plant readiness criteria and a predefined sequence of stages to select plants for release, ensuring that galls are correctly aged to maximize mite numbers. These changes have resulted in substantial improvements in gall abundance (165.3%), *F. perrepae* density per gall (86.0%), and estimated *F. perrepae* per plant (453.2%). The increase in *F. perrepae* released throughout the landscape improved the rates of establishment, abundance, and impact of the agent throughout the invaded range of *L. microphyllum* in Florida.

## Simple Summary

Biological control efforts against the invasive fern *Lygodium microphyllum* in Florida have been significantly enhanced through improvements in mass-rearing techniques for the tiny mite *Floracarus perrepae*, used as a natural enemy (biological control agent) of the fern. In 2008, a release program was initiated for mass-rearing and release of these mites to combat the invasive fern’s spread. In late 2021, improvements in mass-rearing methods led to a substantial increase in mite production. These improvements include the development of a better system for selecting plant readiness, aging the galls (plant growths where the mites reside), and increasing the number of galls and mites per gall. Consequently, large quantities of mites can be released into affected areas, potentially establishing larger populations of the agent. This increase in release capacity may lead to improved control of the invasive fern. This study underscores the importance of refining mass-rearing techniques once an agent is approved for release.

## 1. Introduction

One of the largest hurdles that a new biological control agent must overcome is the establishment threshold [1,2,3]. This is often limited by the ability of practitioners to effectively rear sufficient individuals to meet this threshold. An essential concept of mass-rearing is that these are human-made ecosystems with artificial niches that can be exploited to vastly increase the insect population beyond what would naturally occur [4]. Mass-rearing biocontrol agents can be challenging because each species has complex life history stages, and their specific needs can differ greatly. Successful biocontrol programs maintain invasive populations below critical economic or environmental thresholds, thereby reducing their overall impacts on affected ecological communities [5,6]. Biocontrol agents are often reared with the goal of establishing permanent populations—though augmentative releases are sometimes the strategy employed—in infested areas to recreate evolutionarily coupled population controls on invasive plants. The success of maintaining agent populations in the field can depend critically on the associated mass-rearing program, as its efficacy and output directly contribute to increased release numbers and potentially enhance agent survival and population growth. Refining mass-rearing techniques for biological control agents is crucial for enhancing the effectiveness of invasive species management programs. This study’s improvements in mass-rearing techniques for *Floracarus perrepae* (Acariformes: Eriophyidae), a mite used to help manage the invasive fern *Lygodium microphyllum* (Lygodiaceae; Old World climbing fern), demonstrate how optimizing production methods can dramatically increase agent availability for field releases.

The invasive fern, *L. microphyllum,* causes major environmental and economic damage throughout southern and central Florida [7,8]. With rapid vine-like growth, *L. microphyllum,* frequently produces dense, smothering vegetation mats that climb into tree canopies, where it acts as a fire ladder [7,8,9,10]. The unchecked spread of *L. microphyllum* poses profound risks to the biodiversity of Florida’s ecosystems, with potential economic repercussions for agriculture and tourism [7,8,11,12]. To combat the spread and impact of *L. microphyllum*, an integrated approach has been implemented to control this invasive fern. This strategy incorporates various control methods, including the use of biological control agents. Two such agents have been approved and successfully established: the mite *F. perrepae* and the moth *Neomusotima conspurcatalis* (Lepidoptera: Carambidae).

Given the significant ecological and economic impacts of *L. microphyllum*, this study aims to refine *F. perrepae* mass-rearing techniques to enhance its effectiveness as a biological control agent. *Floracarus perrepae* is an eriophyid mite that stimulates the development of galls both on the margins of the subpinnae of *L. microphyllum* [13], and on the apical meristems, which reduces rachis length and biomass of the vine [12,14]. Galling occurs when the mite feeds on *L. microphyllum* epidermis, causing the cytoplasmic contents of the cells to swell and turn into nutritive tissue [15,16]. A single *F. perrepae* female can initiate the formation of the gall, but as the gall develops, the number of mites increases [13,15,17]. This mite was approved for release in 2006, and the United States Department of Agriculture—Invasive Plant Research Laboratory (IPRL) first released *F. perrepae* in 2008 in Jupiter, Florida [11,18,19]. From 2008 to 2010, small-scale culture and preliminary field releases were conducted with limited establishment at the field sites [19,20]. A mass-rearing and release program was initiated in 2014 as part of the Comprehensive Everglades Restoration Plan, which is a portion of the Congressionally mandated federal/state partnership seeking to mitigate previous anthropogenic damage to this World Heritage ecosystem.

Despite the recognized potential of *F. perrepae* as a biological control agent for the management of *L. microphyllum* [14,21,22], establishment results have been inconsistent [19,20,23]. Studies have demonstrated that release size plays a critical role in the survival and establishment success of biological control agents [1,24]. These findings underscore the need for targeted research to refine mass-rearing protocols for agents like *F. perrepae*. Additionally, it is important to maintain sufficient numbers of an agent for augmented releases following stochastic events such as wildfire, herbicide applications, or extreme temperatures, which may eradicate or substantially diminish the field population [1,2,3,25]. Developing a scalable, efficient, and reproducible mass-rearing protocol for *F. perrepae* is paramount to meeting the growing challenge posed by *L. microphyllum* across Florida. Optimizing the artificial mass-rearing environment of *F. perrepae* can significantly impact its population dynamics and, consequently, possibly its efficacy as a biological control agent in the field.

Prior to this study, mass-rearing protocols for *F. perrepae* lacked standardization, particularly in assessing plant readiness and timing releases with gall age. This research aims to fill these gaps by developing and implementing protocols to enhance mass-rearing efficiency and *F. perrepae* release outcomes. The primary objectives of this study were to (a) develop a standardized method for assessing plant readiness for harvesting, (b) optimize gall aging processes to increase mite/gall density, and (c) assess the effectiveness of these modifications on the productivity of *F. perrepae*.

## 2. Materials and Methods

The first *F. perrepae* colony was obtained from specimens collected in Iron Range, Queensland, Australia [19]. Currently, *F. perrepae* colonies maintained at USDA-ARS-IPRL (26.085° N, −80.240° W) consist of individuals collected from introduced field populations feeding upon *L. microphyllum* in Florida. These colonies occupy roughly 250 m^2^ in screen houses that are maintained at ambient temperatures (averages in the summer 23.3–32.8 °C and winter 13.9–27.2 °C), with shaded natural light. Extreme environmental conditions can be deleterious to the mites [17,18,26]; however, allowing the colony to experience natural fluctuations helps to ensure that they are not thermally limited upon release and thus are more acclimated to conditions found at the release sites [27,28]. The host plant, *L. microphyllum,* has genetic variability that influences susceptibility to galling, so the *F. perrepae* colony was paired with a haplotype that was least resistant to galling [21,29]. The germination methods for *L. microphyllum*, have been relatively unchanged and were described by David and Lake [12]. The new fern maintenance regime calls for Diamond R 90-day slow-release granular fertilizer (12-6-8 NPK, Diamond R Fertilizer, Winter Garden, FL, USA) and Miracle-Gro Water-Soluble All-Purpose Plant Food fertilizers (24-8-16 NPK, Scotts Miracle-Gro Products, Marysville, OH, USA) to be used non-concurrent. Previously, only Diamond R Fertilizer was applied at the recommended dose but at an irregular rate. Diamond R has been observed to cause nutrient burn specifically on the galls and is now applied at the recommended dose after the plants have been cut for release so that no galls are present. To fertilize when galls are present, Miracle-Gro is applied at the recommended dose every two weeks and poses no observed threat to the galls. Excess in phosphorus—which Diamond R has a higher concentration of—can cause nutrient burn to leaves. We suspect galled areas on leaves may be particularly susceptible to this type of stress. This combination ensures that the mites are not negatively affected and the plants retain an adequate amount of nutrients despite the potential for watering outside of our control.

Environmental conditions that we considered in plant growing spaces are the amount of light and the temperature, which must be managed to balance the needs of the plant and the mite [30]. In the case of *L. microphyllum,* plants that receive too much direct light will prematurely produce reproductive fertile pinnae. Additionally, *F. perrepae* struggles to colonize plants in direct sunlight, resulting in a reduction in the number of galls. Alternatively, plants that receive too little sunlight will not be able to keep up with the damage created by the mite, and the plant growth will stall out. Temperature extremes also cause declines in colony populations of *F. perrepae,* which is seen through a reduction in the number of galls and the number of mites per gall. Conversely, *L. microphyllum* grows most quickly during the hottest months of the year.

Since late 2021, we have maintained our mite colony with approximately 4 million individuals as the basis of our release program. While environmental factors play a crucial role in the establishment and spread of biocontrol agents, the genetic diversity within these populations is also important in determining their long-term success. Prior to these changes in mass-rearing, field-collected *F. perrepae* were augmented into the colony biannually to prevent potential genetic bottlenecks [27,28]. These augmentations are no longer employed as the current mass-rearing colony is exponentially larger, and due to concerns about the introduction of additional species of predatory mites.

**Figure 1:**
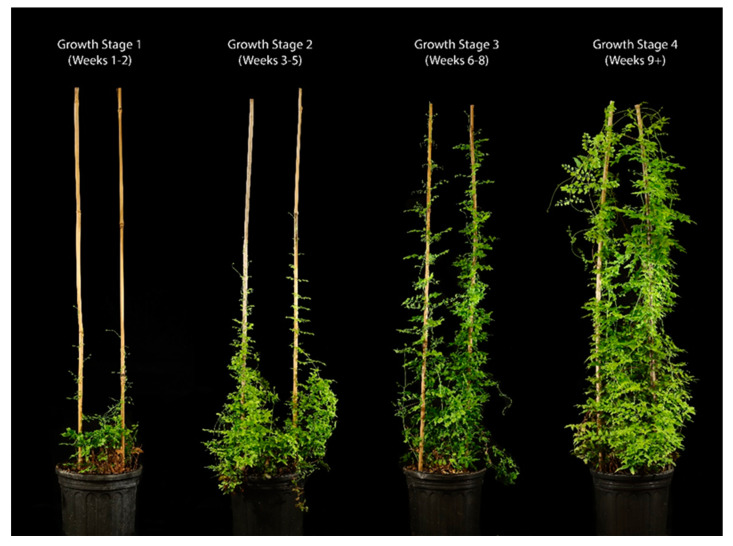
Growth stages 1–4 used to categorize plant growth within the *Floracarus perrepae* colony. Photo credit: Logan Crees.

Rearing protocols that were followed from 2014 to 2021 were a scaled-up version of the maintenance of a colony for experimentation and evaluation in quarantine. These protocols were designed to minimize hands-on care and utilized natural passive transmission of the mites [19]. Plants were haphazardly arranged for mass-rearing and randomly selected for release, with some effort made towards selecting heavily galled plants. In late 2021, the protocols were reevaluated in a pilot study consisting of 24 plants and replaced with more purposeful and labor-intensive management. By the end of 2021, the old protocols were replaced with the new protocols because of the promising increase in average mites per gall in the pilot study plants. These changes relied on a production process that categorized the readiness of plants for release into 4 main stages (Figure 1). These include stage 1 plants ranging from recently trimmed to 30 cm, which takes approximately 2 weeks of growth. Stage 2 is reserved for plants that have rachis around 30 cm or more climbing up bamboo support poles, often taking an additional 3 weeks. Stage 3 consists of plants that have reached the top (60 cm) of the bamboo support poles, which are then allowed to fill out for at least 2 weeks. Stage 4 plants have topped the poles, filled in, have completely expanded pinnae, with most galls in gall age 3 (described below), and are ready to be cut for release. Additionally, plants are targeted for release prior to when the plants switch to allocating more energy towards reproduction [31], and any fertile pinnae are removed. Plants are systematically relocated from one bench to another based on their development stage. Stage 1 plants are positioned at a greater distance from the most severely galled plants (stage 4) to facilitate optimal early plant growth.

We implemented a rotation system where the routine removal of *F. perrepae* through the harvesting of infested plant material, which is then supplemented by complimentary regrowth and/or replacement of *L. microphyllum*. We maintain our *F. perrepae* colony utilizing a continuous contact method, in which infested plants are placed near non-infested *L. microphyllum* plants. The shade house and screenhouse areas typically contain between 400 and 550 *L. microphyllum* plants in total. *Floracarus perrepae* populations are active year-round, peaking in late spring, falling in the summer, then experiencing a smaller increase in fall, with a drop in the winter [17]. During the overlap in the peak growing season for both the *F. perrepae* colony and *L. microphyllum*, host plants are harvested for release every 2 months, with all biomass above 10 cm cut for release. Fertile pinnae are removed before release because *L. microphyllum* utilizes a mixed-mating strategy and is capable of reproducing through intergametophytic processes [32]. During the time of peak overlap, the colony is harvested consistently, approximately every 2 weeks, to keep the population at a level that will not result in overcrowding or collapse of plant quality, as described in Moran et al. [33]. Additionally, we introduce 30 healthy plants, grown in a mite-free laboratory space, every 12 weeks for the replacement of underperforming *L. microphyllum* to sustain the population of *F. perrepae*.

Gall and mite abundances were analyzed from every release where the information had been preserved. Documentation was not always available for the years before the changes were made to the mass-rearing protocols. Typically, these abundances were counted on five haphazardly selected plants and then extrapolated to yield the total estimated mite release numbers using the following equation:

$$\left[\#galls\right]\times \left[\#mites/gall\right]\times \left[\#plants\right]\approx mites\;for\;release.$$

The number of mites/gall was derived by carefully unrolling each gall under a dissecting microscope at 3× magnification and counting the mites as they were exposed. A few releases contained fewer than five plants. For these releases, gall and mite abundances were estimated for all plants following the counting protocols outlined above. On a few occasions, more than five plants per release were assessed. For each year, we calculated the mean and standard error for mites per gall using the mean mite count from each sampling effort.

Through manipulation of host plants, gall-forming organisms induce well-organized galls that are often composed of an inner tissue layer, storage tissue layer, and outer protective layer [34]. In the *L. microphyllum/F. perrepae* system, these galls develop on the margins of sub-pinnae through a series of recognizable phases or “age” classes. We identified five such classes to elucidate the dynamics of mites in their gall microhabitats. Classifying the galls and investigating differences in mite occupancy by gall class was another method used to optimize the selection of plants for release. Class 1 is classified as partially rolled and light green in color; class 2 had one full roll with tissue that is not succulent and light to medium green in color; class 3 had more than one full roll and succulent plant tissue with a medium green color; class 4 had more than one full roll and very succulent plant tissue and a dark green color; and class 5 galls were beginning to necrose, starting in the interior and moving outwards. We estimated the mite density for each gall class by conducting bi-weekly gall dissections from 29 September 2021 to 14 July 2022. Class 5 galls never contained mites and were therefore excluded from the data analysis. The methods used to estimate mite densities per gall class were the same as those outlined above.

Non-parametric tests were chosen due to the non-normal distribution of the data, as indicated by Shapiro–Wilks tests, making the tests listed below more appropriate for our analysis. To assess the effectiveness of the changes to the mass-rearing program, we used Kruskal–Wallis followed by Holm-corrected Dunn tests to compare the average number of galls per plant, the average number of mites per gall, and the estimated number of mites per plant. The Kruskal–Wallis determines if there are statistically significant differences among the years for each of the different parameters. The Dunn’s test is used for pairwise comparisons to identify which specific years differ from each other. The Holm correction was applied to Dunn’s test to control for Type I errors when making multiple pairwise comparisons. All statistical analyses and visualizations were conducted using the R statistical language [35] using the packages ggstatsplot [36] and tidyverse [37]. The release map was created using packages sf [38], mapview [39], raster [40], and leaflet [41].

## 3. Results

### 3.1. Total Floracarus perrepae Released Annually

**Figure 2:**
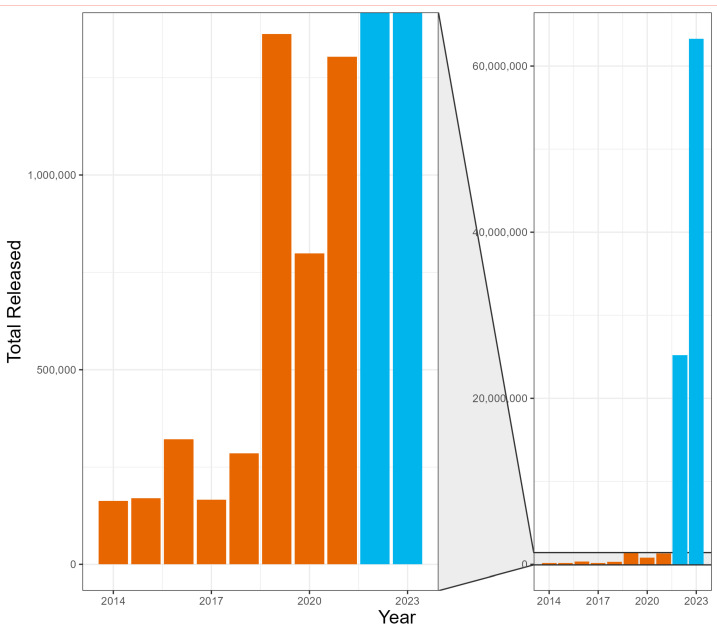
*Floracarus perrepae*, Lygodium mite, release estimates for each year since the mass-rearing and release program started in 2014. The years of the old mass-rearing protocol are shown in orange and the new in blue.

The modified mass-rearing protocols significantly increased *F. perrepae* production. We observed a 46.5-fold increase in the total number of mites released in 2023 compared to the year with the greatest number released prior to modifying the mass-rearing protocols (Figure 2). This increase not only surpassed our projections based on historical trends but also highlights the potential for scaling mass-rearing efforts more effectively. Before the implementation of the new protocols, the mean (±S.E.) mites per release was 23,807 ± 2505, whereas, after the changes, the mean (±S.E.) was 274,976 ± 33,326.

### 3.2. Galls per Plant

**Figure 3:**
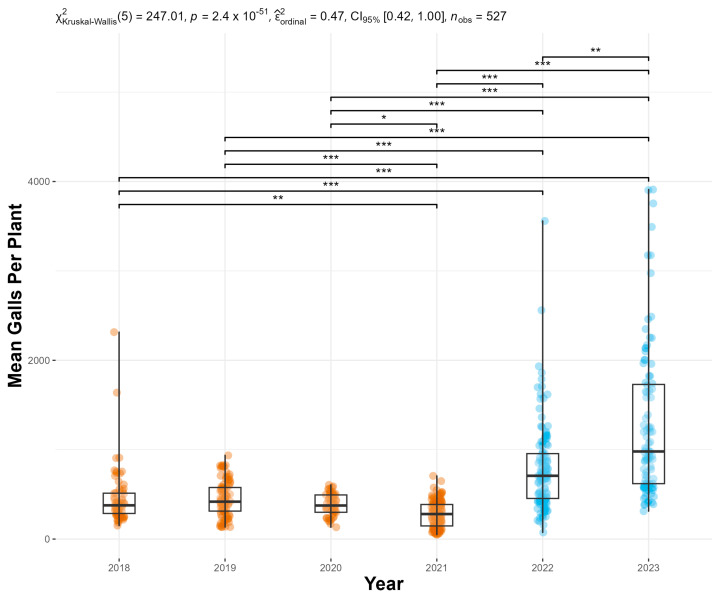
The mean number of galls per plant was calculated from the counts for the five haphazardly selected plants for each release. Significantly different Holm-corrected Dunn pairwise comparisons are shown: * *p* ≤ 0.05, ** *p* ≤ 0.01, *** *p* ≤ 0.001. The years of the old mass-rearing protocol are shown in orange and the new in blue.

From 2018 to 2021, mass-rearing efforts produced 380 ± 12.8 (S.E.) mean galls per plant. Since 2022, we have measured 1008 ± 48.4 (S.E.) mean galls per plant. Thus, our modified mass-rearing protocols produced a 165.3% increase in the mean number of galls on plants harvested for release (Figure 3). The statistical analysis revealed a significant increase in galls post-protocol adjustment (*p* < 0.001), with Figure 3 illustrating a comparison of galls per plant before and after the implementation of the new rearing methods. The increase in galls per plant indicates healthier hosts for *F. perrepae*.

### 3.3. Floracarus perrepae per Gall

**Figure 4:**
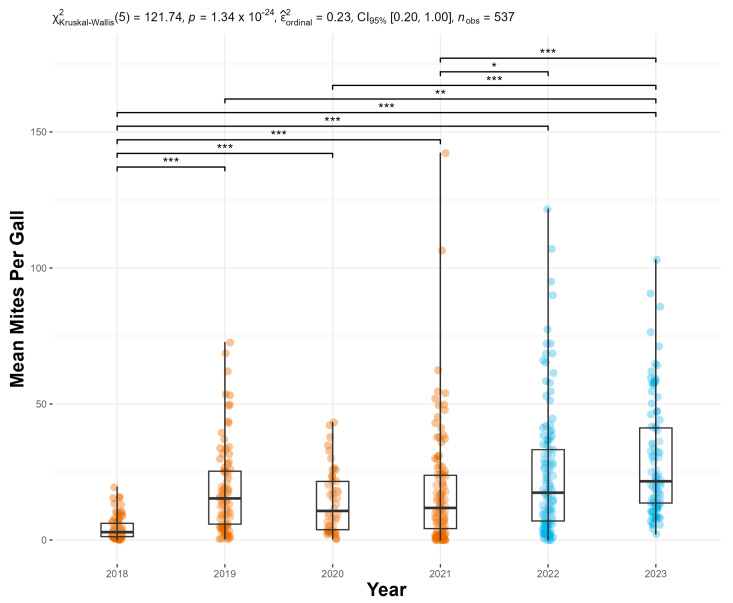
Mean number of *Floracarus perrepae*, Lygodium mites, per gall calculated from dissections of five haphazardly selected galls per plant for each release. Significantly different Holm-corrected Dunn pairwise comparisons are shown; * = *p* ≤ 0.05, ** = *p* ≤ 0.01, *** = *p* ≤ 0.001. The years of the old mass-rearing protocol are shown in orange and the new in blue. All values for year 2021 above 50 are after the change in the protocols.

The implementation of the revised protocols resulted in a substantial increase in mite abundance per gall. Specifically, the mean (±S.E.) mites/gall rose from 14.3 ± 0.931 to 26.6 ± 1.54, representing an 86.0% increase (Figure 4). Notably, the two highest data points for 2021 occurred after the protocol modifications were introduced.

**Figure 5:**
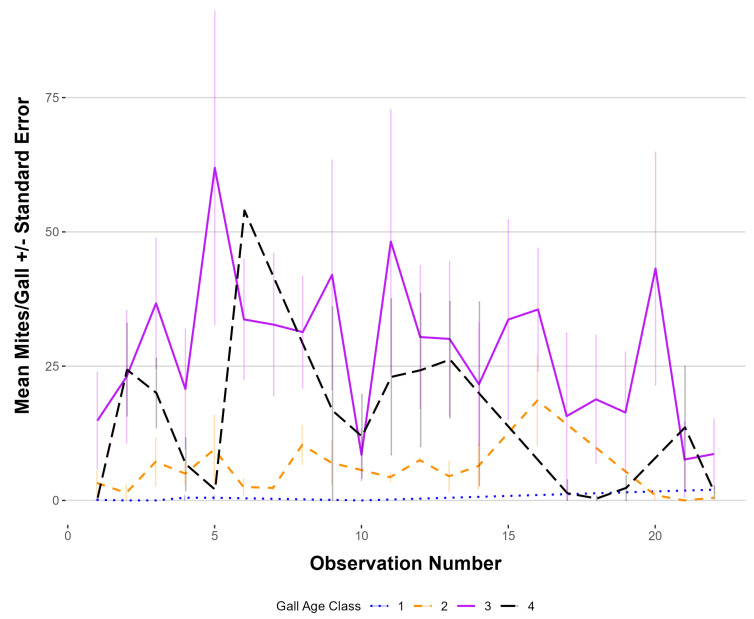
Mean number of *Floracarus perrepae*, Lygodium mites/gall by age class calculated from dissections of five haphazardly selected galls per plant for the biweekly surveys. Standard error bars are represented by the vertical bars.

Gall class 3, on average, consistently had the highest number of mites/gall; therefore, plants are targeted for release when most galls on the plant are in this class (Figure 5). Mite abundance in the galls, regardless of gall class, declined sharply during an extreme heat event in June 2022 (Observation 20). The gall age class 5 was not selected during the survey because, in the preliminary study, no mites were found in these galls.

### 3.4. Floracarus perrepae per Plant

**Figure 6:**
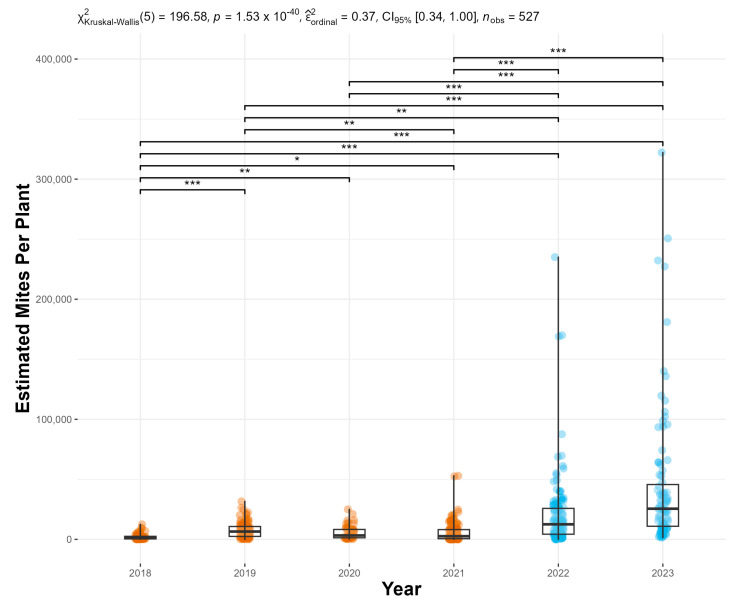
The estimated *Floracarus perrepae*, Lygodium mites, per plant was calculated from the average number of galls per plant and the average number of mites per gall for each release. Significantly different Holm-corrected Dunn pairwise comparisons are shown; * = *p* ≤ 0.05, ** = *p* ≤ 0.01, *** = *p* ≤ 0.001. The years of the old mass-rearing protocol are shown in orange and the new in blue. The two estimated mites per plant above 50,000 came after the changes in the protocols.

The combination of an increase in the number of galls and mites/gall resulted in a 453.2% increase in the estimated mites per plant (Figure 6). The mean (±S.E.) estimated mites/plant was 5659 ± 401 prior to the implementation of the protocol changes, whereas it was 31,308 ± 3141 after the modifications.

### 3.5. Number of Releases

**Figure 7:**
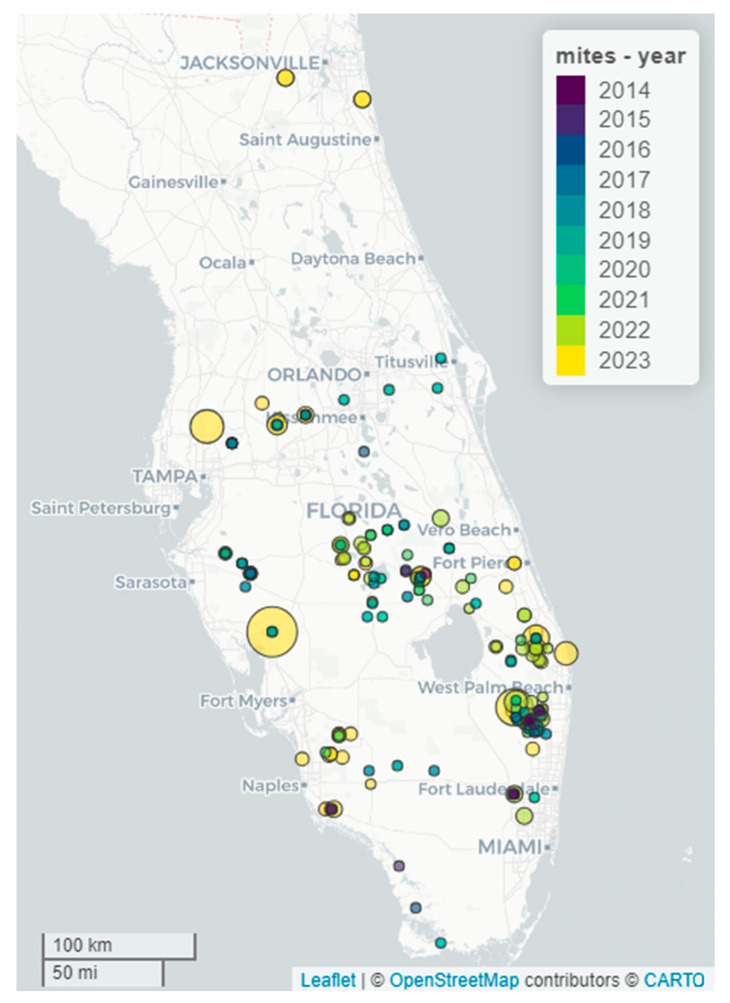
Mite, *Floracarus perrepae*, releases over fiscal years. Circle size indicates relative release size. Circle color indicates the year in which the release occurred. Earlier years are overlaid on more recent years when overlaps occurred. Each release is shown separately even if they occurred in the same year.

During the initial three years (2014–2017) of the mass-rearing program, fewer than seven releases were conducted annually. However, a substantial improvement occurred in 2022–2023, when 43 and 58 releases were conducted, respectively. Not only did the size of the releases have a general trend of greater mite density per plant over time, but the number of plants included in each release also increased, and the number of releases also tended to increase over the years. The annual release data from 2014 to 2023 demonstrates the following progression: 7, 4, 6, 13, 17, 22, 14, 25, 43, and 58 releases (Figure 7). It is important to note that a “release” was defined as an application of biological control agents to a distinct land-managed area, regardless of its acreage or the number of locations within the area where the agents were introduced.

## 4. Discussion

Mass-rearing of the biological control agent *F. perrepae* has challenges, including constructing micro-environments that maintain abiotic conditions as close to optimal as possible for reproduction and galling, as well as maximizing *L. microphyllum* plant vigor, health, regrowth rates, and minimizing pest presence. Release numbers for 2023 were 46.5 times higher than the previous highest year under the old protocols, and this can be attributed to a few key changes in colony maintenance that allowed for greater optimization of the host–parasite population ratio. Finney and Fisher [42] classified mass-rearing as a cost-efficient production that amplifies fecundity by using an assembly line approach while simultaneously reducing the amount of space and time needed for production. This conceptualization guided our approach to implementing changes in mass-rearing protocols, focusing on optimizing efficiency and productivity. We started bi-weekly monitoring of mite numbers within galls and identified the age class (3) at which the most mites were present. Utilizing this information, we created a systematic method (weekly plant readiness criterion based on a predefined sequence of stages) to select infested *L. microphyllum* plants for release that correctly aged galls to maximize mite numbers for release. This assessment of plants increases the probability of early pest detection. This procedure replaced the previous reliance on selecting plants that appeared to have abundant galls with a more systematic process that increased gall numbers (165.3%), mites per gall (86.0%; knowledge of mite density–gall age relationship contributed to this increase), estimated mites per plant (453.2%), releases per year, and enhanced plant vigor. This improvement in production efficiency not only reduces the cost per unit of the biological control agent but may also allow for a more flexible and responsive release strategy.

The potential importance of plant vigor cannot be overstated as it directly relates to oviposition and/or galling success [33,43,44]. Although not directly measured here, plant vigor was associated with color and the fullness of the plant and the fact that plants grew faster, reducing the time in the cycle between harvest and release. The increase in plant vigor likely enhanced *F. perrepae* fecundity by providing more nutritious feeding material, as there is a correlation between host plant health and eriophyid mite reproduction rates [43]. Although increasing plant vigor has proved to be beneficial for *F. perrepae* production, it raises concerns regarding the potential to increase predatory mite population in the galls. Predatory mites have been found to occupy the galls [13,45], and the same factors that increase the mite population are also likely increasing predatory mite populations [46]. Thus, our monitoring of mite numbers and gall age was essential for deducing the optimal time to harvest the plants for releases. Insights gained about optimal gall age and focusing on plant vigor could inform future refinements to mass-rearing techniques for *F. perrepae* and possibly other eriophyid mites. Prior to the adjustments made in our mass-rearing protocols, limitations in production capacity may have contributed to the relatively limited establishment of *F. perrepae* at field sites [19,20,23]. We found that focusing on plant vigor is the most practical means of assessing the rearing potential of *F. perrepae*, as it is completely reliant on its host in all aspects of its lifecycle, and addressing the needs of the plant is better understood than the needs of the microscopic mite. Further justification can be found in a series of studies that show that when mass-rearing, high-quality plants influence biological control agents to produce higher egg yields and divert energy towards reproduction over other pathways [33,43,44]. Aside from assessing the galls they form, discerning the health and fecundity of eriophyid mites is difficult because of their microscopic size and cryptic life [45,47]. This lack of information on the current state of the colony makes it difficult to implement new protocols because the changes in the colony may not be immediately clear. Compromises between the optimal environmental conditions for *F. perrepae* and *L. microphyllum* are required to facilitate the production of the maximum numbers available for release. Within this system, our main conclusion is that to maintain the *F. perrepae* colony for mass-rearing, the focus must be on agronomic practices that increase *L. microphyllum* plant vigor.

The success of a mass-rearing program is evaluated based on its capacity to facilitate the management of the invasive plant, this help in managing the invasive plant is often constrained by the production of a number of biological control agents. We have been extremely successful in mass-rearing *F. perrepae* since implementing changes to the rearing protocols. *Floracarus perrepae*, like many other species of eriophyid mites, utilizes wind-borne dispersal [17,47,48] and, as such, has a relatively high dispersal rate. For example, *F. perrepae* was found at Fakahatchee Strand Preserve State Park in January 2023. The closest known *F. perrepae* release was 28.17 km away at Audubon Corkscrew Swamp Sanctuary, where it had been released on 10 May 2017.

Classical biological control utilizes both inoculative releases, infesting many areas with small numbers of biocontrol agents, and inundative releases, which utilize large releases within small areas [1,49]. Proponents of inundative releases cite concerns that small populations are at greater risk of extinction owing to demographic stochasticity, environmental variability, and Allee effects [2,50]. Our method allows us to hedge bets and essentially marry the two methods by releasing large numbers of individuals at a relatively high number of locations. Additionally, we re-release at sites for several reasons, including where establishment has not occurred, to overcome the effects of predators, where the site is relatively isolated, has experienced climatic extremes, or because they are likely to spread to nearby inaccessible sites.

The improvements in *F. perrepae* mass-rearing productivity demonstrated in this study have important implications for future efforts against *L. microphyllum*, potentially accelerating the establishment and spread of *F. perrepae* in invaded areas. Theoretical and field-based studies have found that releases with high numbers improve the odds of establishment [1,2,3,24,25]. Specifically, our ability to conduct more frequent and larger releases may help overcome establishment thresholds and environmental stochasticity, which can limit successful establishment [1,2,49]. Moreover, our ability to conduct more frequent releases across a wider array of locations not only increases the geographical reach of our biological control efforts but also enhances our ability to target infestations more strategically based on ecological needs and seasonal patterns. The 46.5-fold increase in *F. perrepae* production not only signifies a milestone in improving mass-rearing techniques for this biological control agent but also serves as a critical advancement toward the ecological restoration of habitats invaded by *L. microphyllum*. This increase in mite production potentially improves establishment and provides a cost-effective and ecologically sustainable management method that could reduce reliance on herbicides for the management of *L. microphyllum*. While this study focuses on the increase in mite production and release the broader ecological impacts remain to be fully assessed.

One of the challenges in moving a biocontrol colony from quarantine to mass-rearing is determining how to ramp up production to achieve geometric increases in colony numbers. Our experience shows that paying attention to small details can produce substantial rewards. We achieved this considerable increase in production with standardization of the host plant, focusing on the agent being able to utilize the host plant more efficiently, and increasing host plant vigor. The preservation of host plant vitality over extended periods was not a primary objective of the quarantine protocols, as the plants were used exclusively for the duration of their respective host range tests. This work demonstrates that reevaluating methods when transitioning from maintaining a quarantine colony to implementing mass-rearing can yield positive results, which could be especially beneficial in a field release program.

## Data Availability

The original data presented in the study are openly available in Ag Data Commons at doi.org/10.15482/USDA.ADC/27037888.v2.
